# One or two injections of MVA-vectored vaccine shields hACE2 transgenic mice from SARS-CoV-2 upper and lower respiratory tract infection

**DOI:** 10.1073/pnas.2026785118

**Published:** 2021-03-09

**Authors:** Ruikang Liu, Jeffrey L. Americo, Catherine A. Cotter, Patricia L. Earl, Noam Erez, Chen Peng, Bernard Moss

**Affiliations:** ^a^Laboratory of Viral Diseases, National Institute of Allergy and Infectious Diseases, NIH, Bethesda, MD 20892

**Keywords:** COVID-19, coronavirus vaccine, modified vaccinia virus Ankara, neutralizing antibody, transgenic mouse model

## Abstract

Modified vaccinia virus Ankara (MVA) is a replication-restricted smallpox vaccine, and numerous clinical studies of recombinant MVAs (rMVAs) as vectors for prevention of other infectious diseases, including COVID-19, are in progress. Here, we characterize rMVAs expressing the S protein of severe acute respiratory syndrome coronavirus 2 (SARS-CoV-2). Modifications of full-length S individually or in combination included two proline substitutions, mutations of the furin recognition site, and deletion of the endoplasmic retrieval signal. Another rMVA in which the receptor binding domain (RBD) is flanked by the signal peptide and transmembrane domains of S was also constructed. Each modified S protein was displayed on the surface of rMVA-infected cells and was recognized by anti-RBD antibody and soluble hACE2 receptor. Intramuscular injection of mice with the rMVAs induced antibodies, which neutralized a pseudovirus in vitro and, upon passive transfer, protected hACE2 transgenic mice from lethal infection with SARS-CoV-2, as well as S-specific CD3+CD8+IFNγ+ T cells. Antibody boosting occurred following a second rMVA or adjuvanted purified RBD protein. Immunity conferred by a single vaccination of hACE2 mice prevented morbidity and weight loss upon intranasal infection with SARS-CoV-2 3 wk or 7 wk later. One or two rMVA vaccinations also prevented detection of infectious SARS-CoV-2 and subgenomic viral mRNAs in the lungs and greatly reduced induction of cytokine and chemokine mRNAs. A low amount of virus was found in the nasal turbinates of only one of eight rMVA-vaccinated mice on day 2 and none later. Detection of low levels of subgenomic mRNAs in turbinates indicated that replication was aborted in immunized animals.

## Significance

Vaccines are required to control COVID-19 during the current pandemic and possibly afterward. Recombinant nucleic acids, proteins, and virus vectors that stimulate immune responses to the SARS-CoV-2 S protein have provided protection in experimental animal and human clinical trials, although questions remain regarding their ability to prevent spread and the duration of immunity. The present study focuses on replication-restricted modified vaccinia virus Ankara (MVA), which has been shown to be a safe, immunogenic, and stable smallpox vaccine and a promising vaccine vector for other infectious diseases and cancer. In a transgenic mouse model, one or two injections of recombinant MVAs that express modified forms of S inhibited SARS-CoV-2 replication in the upper and lower respiratory tracts and prevented severe disease.

Recombinant DNA methods have revolutionized the engineering of vaccines against microbial pathogens, thereby creating opportunities to control the current COVID-19 pandemic (1). The main categories of recombinant vaccines are protein, nucleic acid (DNA and RNA), virus vectors (replicating and nonreplicating), and genetically modified live viruses. Each approach has advantages and drawbacks with regard to manufacture, stability, cold-chain requirements, mode of inoculation, and immune stimulation. Recombinant proteins have been successfully deployed as vaccines against a variety of diseases (2–5). DNA vaccines have been licensed for veterinary purposes (6, 7), although none are in regular human use. Recently developed messenger RNA (mRNA) vaccines are in use for COVID-19 and are in preclinical development for other infectious diseases (8). At least 12 virus vector vaccines based on adenovirus, fowlpox virus, vaccinia virus (VACV), and yellow fever virus have veterinary applications, but, so far, only two have been marketed for humans (9), although numerous clinical trials, particularly with attenuated adenovirus and VACV, are listed online in ClinicalTrials.gov.

A variety of recombinant approaches utilizing the spike (S) protein of severe acute respiratory syndrome coronavirus 2 (SARS-CoV-2; abbreviated CoV-2) as immunogen are being explored to quell the COVID-19 pandemic (10). Vaccines based on mRNA and adenovirus vectors have demonstrated promising results in clinical trials and have received emergency regulatory approval (11–14). Other candidate CoV-2 vaccines, including ones based on vesicular stomatitis virus (15), an alphavirus-derived replicon RNA (16), an inactivated recombinant Newcastle Disease virus (17), and modified VACV Ankara (MVA) (18, 19) are at early stages of evaluation.

Experiments with virus vectors for vaccination were carried out initially with VACV (20, 21), providing a precedent for a multitude of other virus vectors (9). The majority of current VACV vaccine studies employ the MVA strain, which was attenuated by more than 500 passages in chicken embryo fibroblasts during which numerous genes were deleted or mutated, resulting in an inability to replicate in human and most other mammalian cells (22). Despite the inability to complete a productive infection, MVA is capable of highly expressing recombinant genes and inducing immune responses (23, 24). MVA is a licensed smallpox vaccine, and numerous clinical studies of recombinant MVA (rMVA) vectors are in progress or have been completed. Protection has been obtained with MVA-based SARS-CoV-1 and Middle East respiratory syndrome CoV (MERS-CoV) in animals (25–28), and an MVA-based MERS-CoV vaccine was shown to be safe and immunogenic in a phase 1 clinical trial (29). Currently, two clinical trials for MVA-based CoV-2 vaccines are in the recruiting phase (ClinicalTrials.gov). Here, we show that one or two immunizations with rMVAs expressing the CoV-2 S proteins elicit strong neutralizing antibody responses, induce CD8+ T cells, and protect susceptible transgenic mice against a lethal intranasal challenge with CoV-2 virus, supporting clinical testing of related rMVA vaccines.

## Results

### Construction of rMVAs that Express Modified CoV-2 S Proteins.

**Fig. 1.**
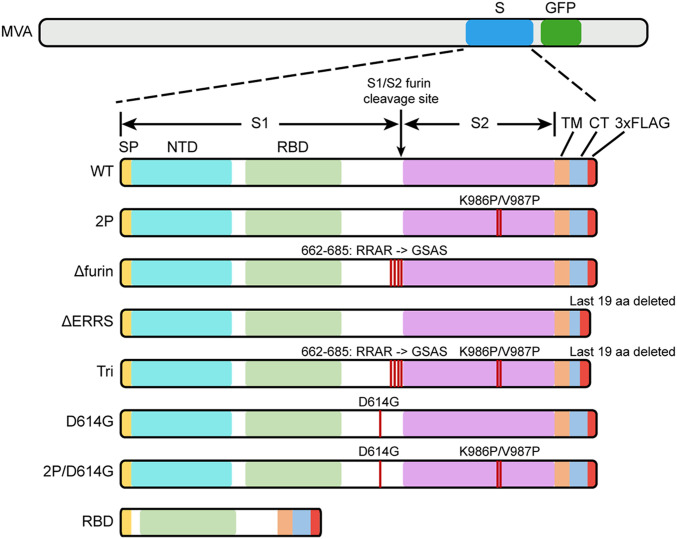
Diagrams of rMVAs. Top shows approximate locations of CoV-2 S protein (S) and GFP open reading frames (ORFs) within rMVA. Modifications of S ORF are shown below, with names of constructs on the left. SP, signal peptide; NTD, N-terminal domain; TM, transmembrane domain; CT, C-terminal domain; 3×FLAG, three tandem copies of FLAG epitope tag.

The full-length CoV-2 S protein contains 1,273 amino acids (aa) comprising a signal peptide (aa 1 to 13), the S1 receptor binding subunit (aa 14 to 685), and the S2 membrane fusion subunit (aa 686 to 1,273). A panel of rMVAs with unmodified and modified versions of CoV-2 S (GenBank: QHU36824) with C-terminal FLAG tags were engineered by homologous recombination (Fig. 1). The rMVAs, which are designated in the text by abbreviated italicized names, include *WT* with unmodified S, *2P* with two proline substitutions (K_986_P, V_987_P) intended to stabilize the prefusion conformation (11, 30–32), *Δfurin* with perturbation of the furin recognition site (RRAR_682–685_GSAS) to prevent cleavage of S, *ΔERRS* with deletion of the last 19 aa including the endoplasmic reticulum retrieval signal, and *Tri* with a combination of all three modifications. *RBD*, another rMVA, contains the receptor binding domain (RBD) and contiguous sequences preceded by the S signal peptide and followed by the transmembrane domain of S. The latter features were added because a previous study with an unrelated protein demonstrated that membrane anchoring strongly enhances immunogenicity of a VACV vector (33) and also enhances immunogenicity of CoV-2 S expressed by an mRNA vaccine (11). The additional rMVAs, *D*_*614*_G and a *2P* version, express S with amino acid changes of a variant CoV-2 strain (34). VACV transcription termination signals that could reduce early expression (35) and runs of four or more consecutive Gs or Cs that could accelerate the occurrence of deletions (36) were altered by making silent mutations.

**Fig. 2.**
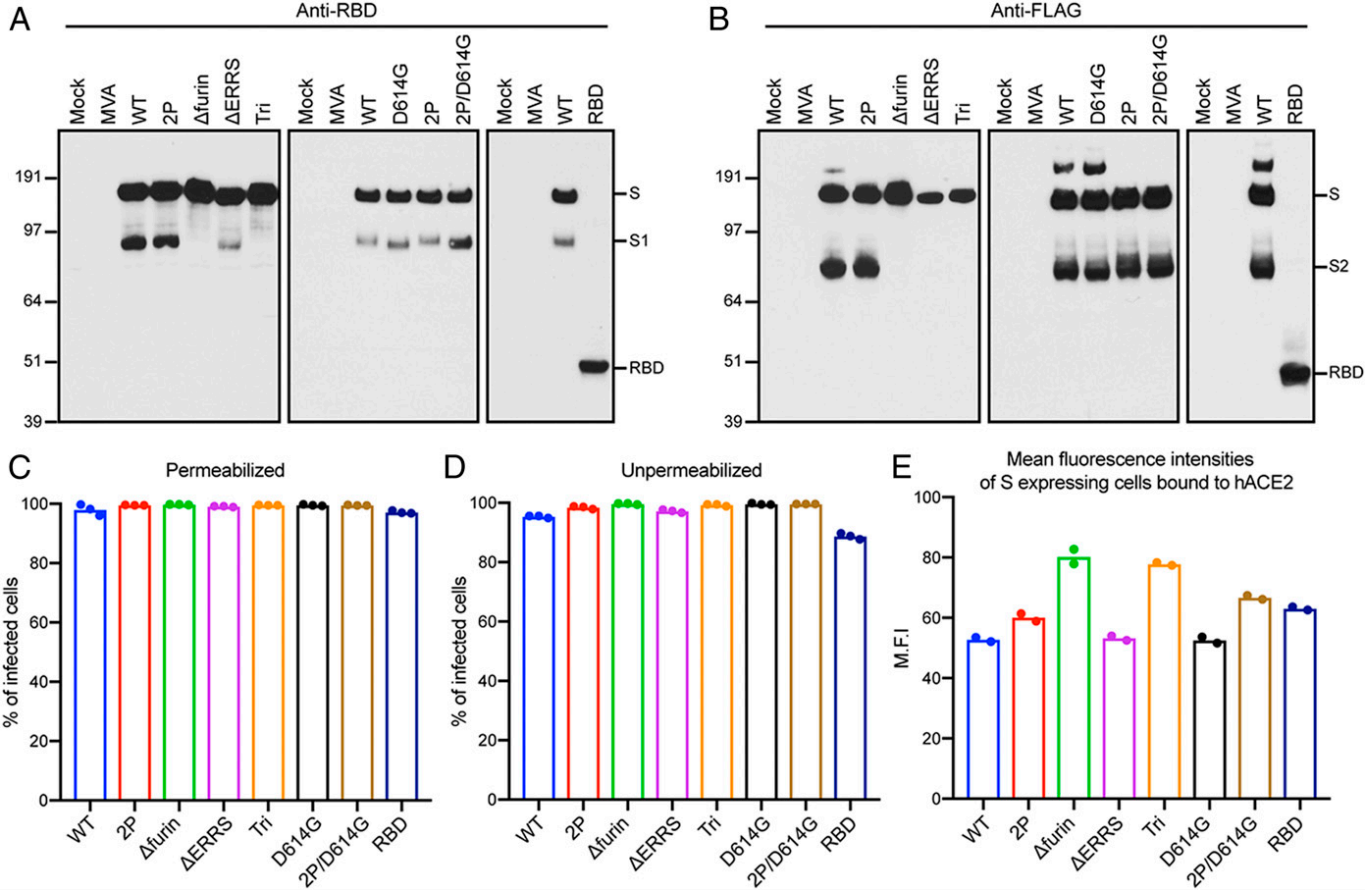
Expression of modified S proteins. (*A* and *B*) Western blots. HeLa cells were mock infected or infected with 5 PFU per cell of purified parental MVA or rMVAs for 18 h, and lysates were analyzed by SDS-polyacrylamide gel electrophoresis. Because all samples were not analyzed in the same gel, WT rMVA was included in each as a positive control. After membrane transfer, the proteins were detected with antibody to RBD or FLAG. The positions and masses, in kilodaltons, of marker proteins are indicated on the left, and the positions of S, S1, S2, and RBD are indicated on right. Representatives of three repeats are shown. (*C* and *D*) Flow cytometry. HeLa cells were infected in triplicate and permeabilized or stained directly with anti-SARS-CoV-2 Spike RBD mAb followed by APC-conjugated goat anti-mouse IgG. Infected cells were identified by GFP fluorescence, and the percent that express S was determined. Bars represent the geometric mean. A representative of two independent experiments is shown. (*E*) Mean fluorescent intensities. HeLa cells were infected in duplicate and incubated with soluble hACE2 followed by Alexa Fluor 647-conjugated anti-hACE2 antibody. Cells that express S were identified as in the previous panels, and the intensity of anti-hACE2 antibody was determined. A representative of two independent experiments is shown.

Human HeLa cells were infected with purified rMVAs, and cell lysates were analyzed by sodium dodecyl sulfate (SDS)-polyacrylamide gel electrophoresis and Western blotting. Full-length S proteins of ∼180 kDa, or a ∼50-kDa shortened version in the case of *RBD*, were detected by antibodies to the RBD (Fig. 2*A*) and to the C-terminal FLAG tag (Fig. 2*B*). The anti-RBD antibody also recognized S1, formed by cleavage of full-length S, from lysates of cells infected with *WT* (wild type),* D*_*614*_G, and their *2P* versions but only a small amount from *ΔERRS* and none from *Δfurin* or *Tri*, both of which have deletions of the furin recognition site (Fig. 2*A*). Similarly, S2 was detected by anti-FLAG antibody in lysates from cells infected with *WT*, *D*_*614*_G, and their *2P* versions but not from *Δfurin*- or *Tri* (Fig. 2*B*). The relatively low intensities of the *ΔERRS* and *Tri* bands when probed with anti-FLAG compared to anti-RBD suggests reduced accessibility of the former antibody due to the C-terminal truncation. Small amounts of more slowly migrating S, possibly representing undissociated trimers or larger aggregates, were also detected by anti-FLAG antibody in cells infected with *WT* and *D614G* (Fig. 2*B*).

### Modified CoV-2 S Proteins Are Expressed on the Surface of Human Cells.

Expression of the CoV-2 S proteins in HeLa cells that were infected with the rMVAs was evaluated by flow cytometry. After permeabilization, virtually 100% of infected cells distinguished by green fluorescent protein (GFP) fluorescence were stained by a mouse anti-RBD mAb as shown in scatter plots and histograms (*SI Appendix*, Fig. S1) and summarized in Fig. 2*C*. In the absence of permeabilization, nearly 100% of the cells expressing full-length S and nearly 90% expressing the RBD were stained, indicating cell surface expression (*SI Appendix*, Fig. S2 and Fig. 2*D*). Control experiments with unmodified parental MVA demonstrated no significant staining with or without permeabilization.

Human angiotensin converting enzyme (hACE2) is a cell receptor for CoV-2 (1, 37). The binding of soluble hACE2 to S proteins expressed on the surface of cells infected with rMVAs was analyzed as an indication of their appropriate folding. Binding of hACE2 to all constructs is shown in histograms (*SI Appendix*, Fig. S3). The mean fluorescence intensities of S-expressing cells were similar except for the slightly higher value with *Δfurin* and *Tri* (Fig. 2*E*). We concluded that the WT and modified S proteins were all highly expressed on the surface of infected HeLa cells and potentially capable of eliciting immune responses.

### rMVAs Induce CoV-2 S-Binding and Neutralizing Antibodies.

**Fig. 3.**
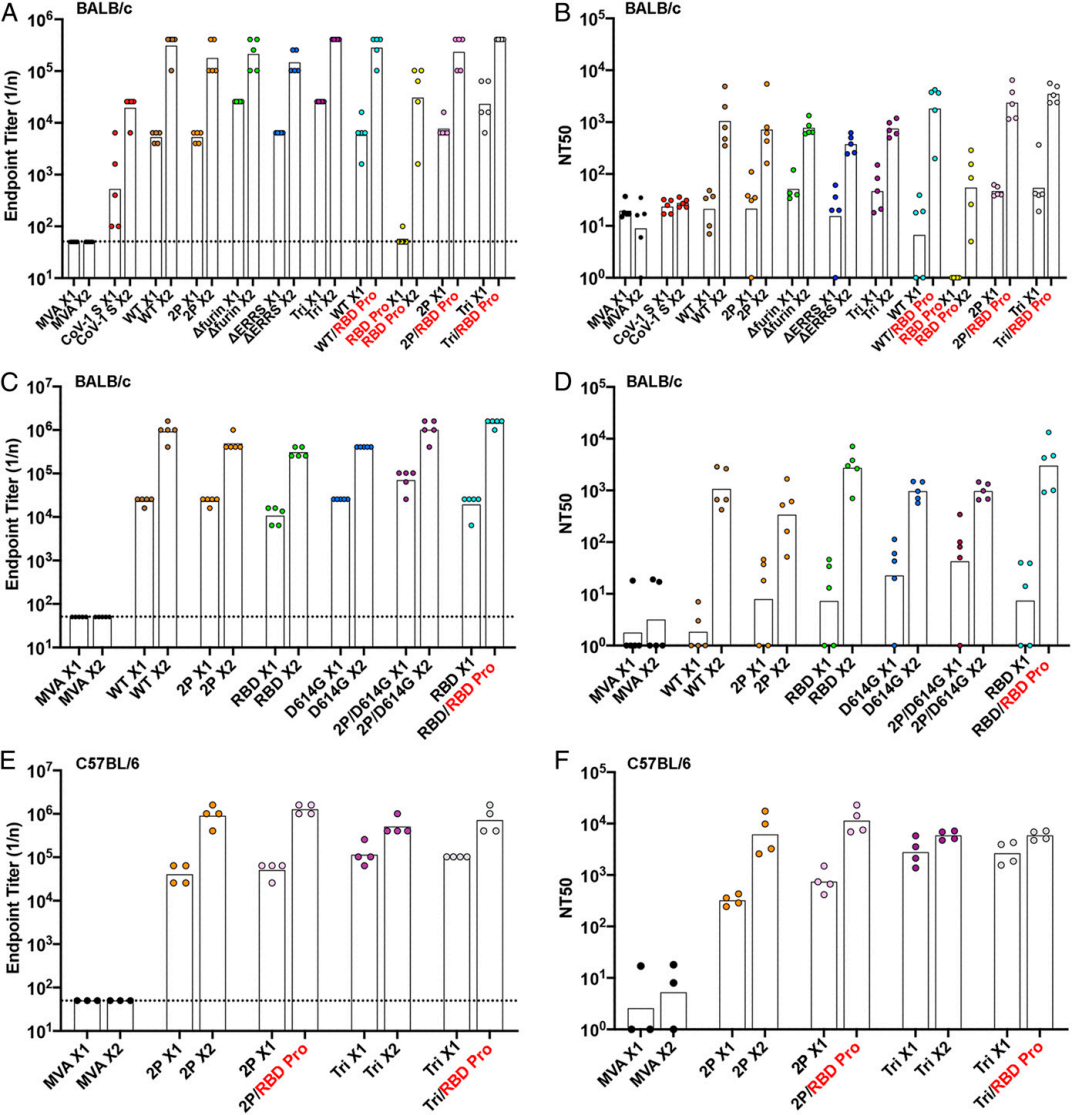
Binding and neutralizing antibody responses. (*A*–*D*) BALB/c or (*E* and *F*) C57BL/6 mice were primed and boosted 3 wk later with 2 × 10^7^ PFU of parental MVA, rMVA expressing SARS-CoV-1 S, rMVA expressing WT, or modified CoV-2 S, or with 10 µg of RBD protein in QS21 adjuvant. Mice were bled before vaccination, at 3 wk after the prime, and at 2 wk after the boost. Antibody binding to S was determined by ELISA. Reciprocal end point binding titers are shown in *A*, *C* and *E*. Dotted lines indicate limit of detection. Pseudovirus neutralization titers are shown in *B*, *D* and *F* and plotted as NT50. The tops of bars are geometric mean titers. X1, sera collected after prime; X2, sera collected after homologous boost; heterologous boost with RBD protein (RBD Pro in red). Data are representatives of eight experiments in which binding and neutralizing antibodies were determined. The means of the groups were compared by ANOVA using the nonparametric Kruskal−Wallis test with Dunn’s multiple comparisons.

To evaluate immunogenicity, 2 × 10^7^ plaque-forming units (PFU) of each purified rMVA was inoculated intramuscular(ly) (IM) into BALB/c mice at 0 time and again at 3 wk. In addition, RBD protein (10 µg) in QS21 adjuvant was administered IM to some mice for priming and boosting or for boosting after priming with an rMVA. Binding antibody was measured by enzyme-linked immunosorbent assay (ELISA) using wells coated with purified 2P-stabilized S protein. End point titers of sera from BALB/c mice immunized with the vectors constructed earlier (*WT*, *2P*, Δ*furin*, Δ*ERRS*, and *Tri*) and later (*RBD*, *D614G*, and *2P/D614G*) plus repeats of *WT* and *2P* are shown in Fig. 3 *A* and *C*, respectively. Binding antibodies were detected 3 wk after the first immunization and increased by more than one log at 2 wk following a boost with the homologous rMVA. Lesser binding to CoV-2 S, representing cross-reactivity, was obtained with sera from mice immunized with rMVA expressing the SARS-CoV-1 S (Fig. 3*A*), and no binding above the base line was detected with sera from mice immunized with the parental MVA lacking S sequences (Fig. 3 *A* and *C*). Sera from mice immunized with adjuvanted RBD protein exhibited low or no binding to S after the first inoculation and less binding after the second than sera from mice immunized with rMVAs (Fig. 3*A*). Nevertheless, the RBD protein effectively boosted antibody in mice primed with rMVAs (Fig. 3 *A* and *C*). The inability of RBD protein to induce binding antibody in naive mice was probably due to low immunogenicity of the soluble protein, since the titer obtained after one vaccination with the rMVA *RBD,* which encodes a membrane-bound version of the protein, was similar to titers obtained with full-length S (Fig. 3*C*).

Neutralizing titers of serum from BALB/c mice were determined using a previously described lentiviral pseudotype assay (11, 38). Low neutralizing activity was detected at 3 wk after priming with rMVAs expressing CoV-2 S, but increased greatly at 2 wk after homologous rMVA boosts to mean levels of ∼10^3^ NT50 (reciprocal of the serum dilution for 50% reduction in luciferase activity) (Fig. 3 *B* and *D*). Serum from mice infected with the rMVA expressing SARS CoV-1 S did not neutralize CoV-2 even though binding to full-length S occurred. The RBD protein generally boosted NT50 titers higher than the rMVAs, although the difference was not always of high statistical significance. Three samples of patient sera that had reference CoV-2 50% neutralizing titers of 1,280, 320, and 320 were found to have pseudovirus NT50 titers of 3,209, 370, and 482, respectively. Thus, the rMVAs produced neutralizing antibody that was in the high range for patient sera.

It was of interest to compare the immunogenicity of the rMVAs in BALB/c mice with C57BL/6 mice, which induce Th2- and Th1-prone responses, respectively. MVA neutralizing antibodies were detected in serum from both strains of mice after the prime and boost (*SI Appendix*, Fig. S4). The S-binding antibody titers of *2P* and *Tri* were significantly higher in sera from C57BL/6 mice than BALB/c mice after the prime (*P* < 0.004) but were similar after the boosts (compare Fig. 3*E* with Fig. 3 *A* and *C*). The CoV-2 neutralizing titers obtained by immunization with *2P* and *Tri* also were significantly higher (*P* < 0.001) for the prime in C57BL/6 (Fig. 3*F*) than in BALB/c (Fig. 3 *B* and *D*) mice, whereas the increase was less significant for the boosts (*P* < 0.02).

A time course indicated that binding and neutralizing antibodies were detected after 1 wk and increased greatly by 3 wk after the first immunization of C57BL/6 mice (*SI Appendix*, Fig. S5). An additional experiment showed that soluble S proteins and RBD from a variety of sources adjuvanted with QS-21 boosted binding and neutralizing antibodies in C57BL/6 mice (*SI Appendix*, Fig. S6).

### rMVAs Induce Predominantly IgG2a and IgG2c S-Specific Antibodies.

We determined the IgG subclasses of CoV-2 S-specific antibodies induced by an MVA-based vector and by QS21-adjuvanted RBD protein by a sandwich ELISA. Table 1 shows that BALB/c and C57BL/6 mice that were primed and boosted by *2P* made IgG1, IgG2b, IgG2a, or IgG2c and IgG3, but no detectable IgA antibody. The highest values were IgG2a and IgG2c in BALB/c and C57BL/6, respectively, which was consistent with a predominant Th1 response (39–41). The isotypes produced in hACE2 mice, which were backcrossed to C57BL/6 and used in a later section of the paper, were similar to that of C57BL/6. However, the biggest difference was between the RBD protein prime and boost and immunizations with rMVA (Table 1). The protein immunization elicited a predominance of IgG1, giving a clear Th2 response. Nevertheless, the RBD protein following rMVA boosted the Th1 response in both C57BL/6 and BALB/c mice.

**Table 1.** Isotype analysis of anti-S antibodies

			10^3^ Reciprocal end point titer*					IgG2a,c/IgG1 ratio	
Strain	Vaccine	IgA	IgG1	IgG2a^†^	IgG2b	IgG2c^‡^	IgG3	IgG2a/IgG1	IgG2c/IgG1
BALB/c	*2P* × 2^§^	ND^¶^	102	1,005	26	—	4	9.85	—
	*2P*/RBD^#^	ND	102	1,600	64	—	6	15.69	—
	RBD/RBD^‖^	ND	102	16	2	—	0	0.16	—
									
C57BL/6	*2P* × 2^§^	ND	16	—	102	409	4	—	25.56
	*2P*/RBD^#^	ND	26	—	102	1,005	6	—	39.24
									
K18-hACE2	*2P* × 2^§^	ND	26	—	102	409	2	—	15.98
	*2P/*RBD^#^	ND	26	—	256	409	6	—	15.98

* Mean of duplicates of pooled sera.

^†^ Gene not present in C57BL/6 and K18-hACE2 mice.

^‡^ Gene not present in BALB/c mice.

^§^ The *2P* followed by *2P*.

^¶^ ND, not detected.

^#^ The *2P* followed by RBD protein.

^‖‖^ RBD protein followed by RBD protein.

### rMVAs Stimulate Production of S-Specific CD3+CD8+IFNγ+ T Cells.

**Fig. 4.**
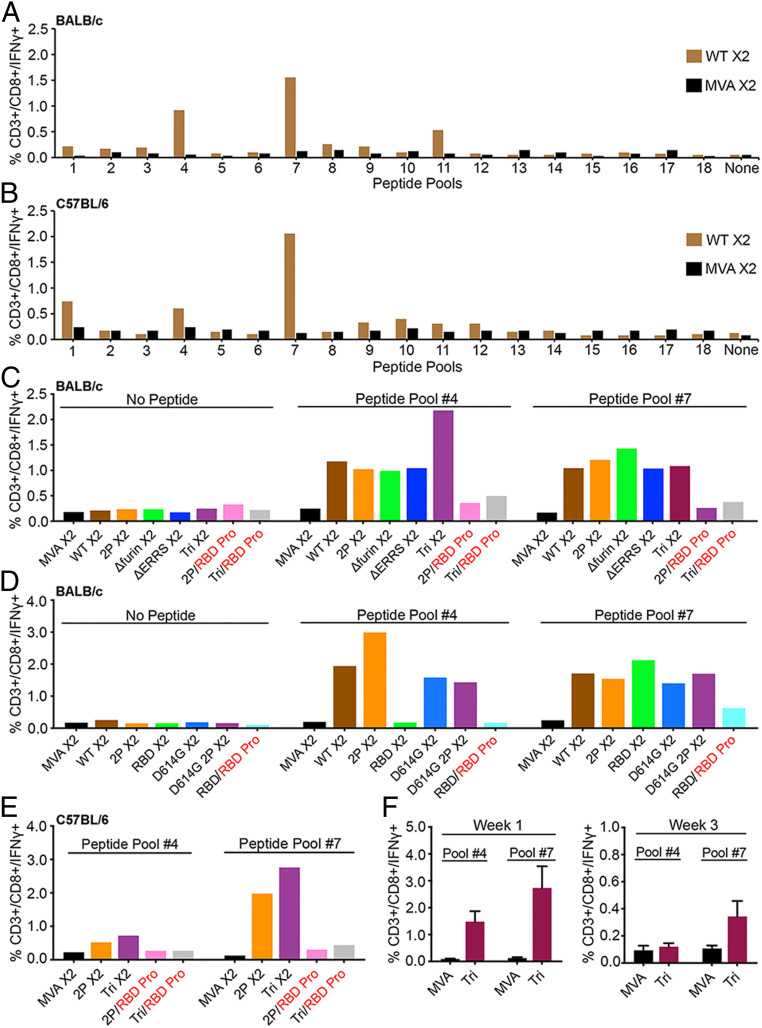
CD8+ T cell responses. (*A*) BALB/c mice and (*B*) C57BL/6 mice were vaccinated IM with 2 × 10^7^ PFU of unmodified MVA or rMVA expressing WT CoV-2 S at 0 time and again after 3 wk. At 2 wk after the boost, spleen cells were pooled from three to five mice and stimulated with pools of peptides derived from CoV-2 S protein and treated with Brefeldin A. Cells were then stained for cell surface markers with mouse anti-CD3-FITC, anti-CD4-PE, and anti-CD8-PerCP. Cells were subsequently stained intracellularly with mouse anti-IFNγ-APC. CD3+CD8+IFNγ+ cells were enumerated by flow cytometry. (*C* and *D*) BALB/c and (*E*) C57BL/6 mice were primed with the indicated parental MVA or rMVA and boosted with the homologous rMVA or with RBD protein. Splenocytes from four to five mice were combined and stimulated with pool #4 and pool #7 peptides and then analyzed as in *A* and *B*. (*F*) C57BL/6 mice were primed with parental MVA or rMVA *Tri*. After 1 and 3 wk, the splenocytes of individual mice (*n* = 4) were analyzed as in *A*. SDs are shown. X2 refers to splenocytes collected after homologous rMVA boost; RBD Pro in red indicates heterologous boost with RBD protein.

An ex vivo stimulation protocol identified T cells specific for S following immunization. The sequences of an array of CoV-2 S peptides obtained from BEI Resources were compared to peptides that were previously found positive for BALB/c mice (42). As peptides were not identical in the two libraries, we tested pools for their ability to stimulate CD3+CD8+IFNγ+ T cells from spleens of BALB/c mice that had been immunized by priming and boosting with parental MVA or rMVA *WT* expressing CoV-2 S. The two S peptide pools with highest specific activity were #4 and #7, which contained peptides from the NTD and RBD portions of S1, respectively (Fig. 4*A*). The pools were also tested for ability to stimulate CD3+CD4+IFNγ+ specific T cells, but none exhibited strong activity. A similar screen was carried out with spleen cells derived from immunized C57BL/6 mice. Pool #7 was again most positive for CD8+IFNγ+ T cells, whereas other pools showed less activity (Fig. 4*B*).

Next, we compared the percentages of splenic CD8+IFNγ+ T cells following priming with our first and second sets of rMVA S constructs followed by homologous rMVA or RBD protein boosts (Fig. 4 *C* and *D*). Spleen cells from mice immunized with parental MVA lacking S sequences and spleen cells that were not stimulated with peptide served as negative controls. A representative gating strategy for CD3+CD8+IFNγ+ T cells is shown in *SI Appendix*, Fig. S7. RBD peptides (pool #7) stimulated CD8+IFNγ+ T cells from mice immunized by priming and boosting with each of the rMVA S constructs (Fig. 4 *C* and *D*). A similar result was obtained with NTD peptides (pool #4), except for the absence of stimulation of CD8+IFNγ+ T cells from mice immunized with *RBD*, which lacks those sequences. Mice that were primed with an rMVA and boosted with RBD protein had low levels of CD8+IFNγ+ T cells (Fig. 4 *C* and *D*). A similar result occurred in C57BL/6 mice: The CD8+IFNγ+ T cells were higher after rMVA boosts than after RBD protein boosts (Fig. 4*E*). This difference appears to be due to a rapid decline of CD8+IFNγ+ T cell numbers following the rMVA prime and an inability of the RBD protein to restimulate them. In accord with this idea, a substantial drop in CD8+IFNγ+ T cell numbers occurred between 1 and 3 wk after immunization with an rMVA (Fig. 4*F*).

### Immunization with rMVAs Protects Transgenic Mice from CoV-2.

**Fig. 5.**
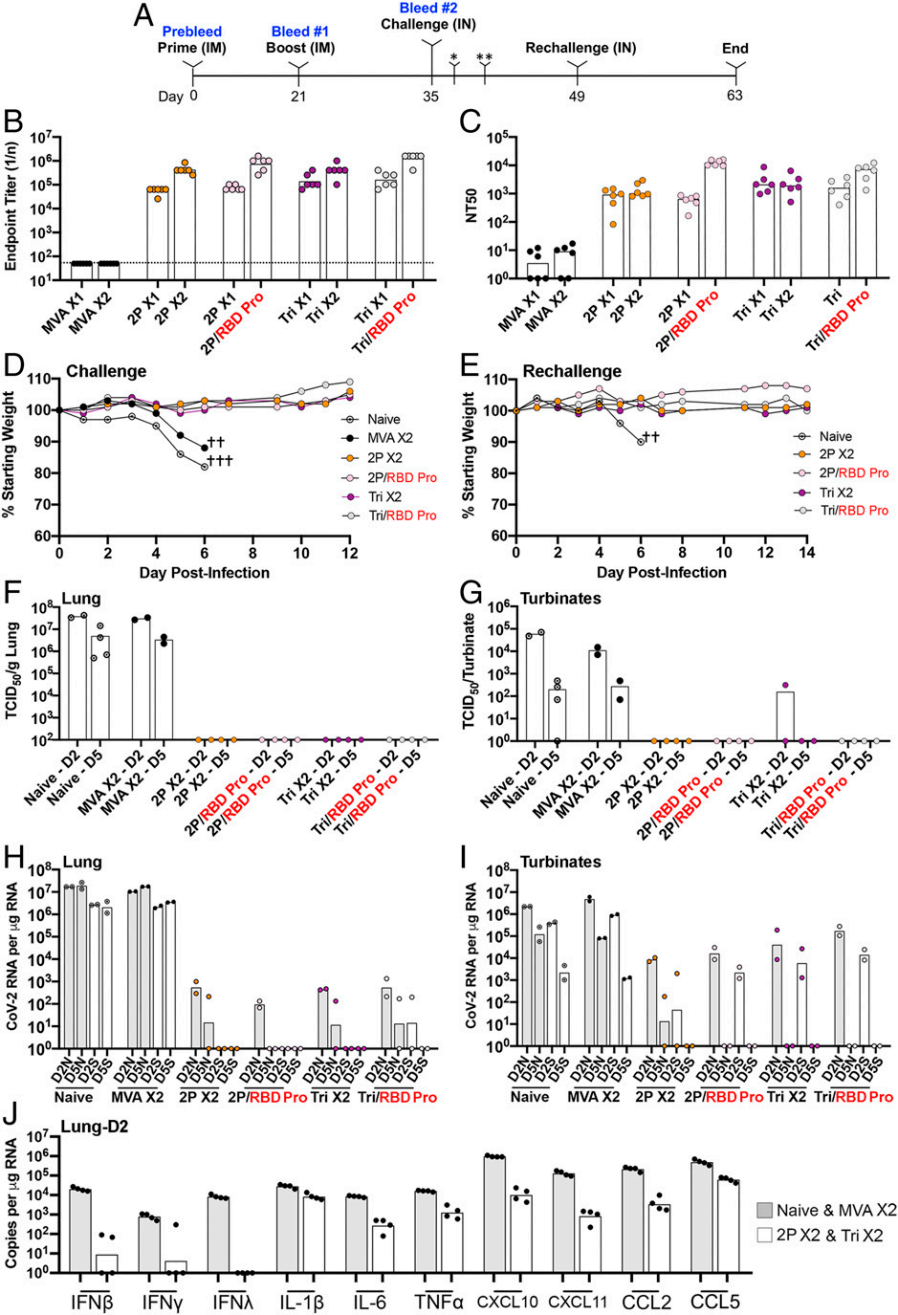
Challenge of transgenic mice following prime and boost vaccinations. (*A*) Thirty K18- hACE2 mice (female, 7 wk old) were vaccinated IM with 2 × 10^7^ PFU of parental MVA or rMVA on days 0 (prime) and 21 (boost), and six remained unvaccinated. Six of the vaccinated mice were inoculated with parental MVA, two groups of six with *2P*, and two groups of six with *Tri*. One *2P* and one *Tri* group were boosted with the homologous vector and the other with RBD protein. Mice were bled on days 0, 21, and 35 to determine S binding and CoV-2 neutralizing titers. Naive mice and vaccinated mice 2 wk after boosting were infected IN with 10^5^ TCID50 of CoV-2. Mice were followed for weight loss and signs of morbidity. Two mice from each group were euthanized on days 2 (*) and 5 (**) after challenge to determine infectious CoV-2 virus and subgenomic mRNA. A second IN challenge of surviving mice and two added naive mice was performed 2 wk after the first challenge. (*B*) Binding antibody was determined by ELISA and plotted as 1/end point dilution. Dotted line indicates limit of detection. (*C*) Neutralizing antibody was determined by a pseudovirus assay and plotted as NT50. (*D*) Weights of surviving mice were determined daily and plotted as percent of starting weight. Symbol † indicates number of mice that died or were euthanized on a specific day. Data for one naive mouse was from a preliminary experiment. (*E***)** Weights were determined after the rechallenge of surviving mice and two naive mice. (*F*) Virus titers in lung homogenates obtained on days 2 and 5 were determined by end point dilution and plotted as TCID50 per gram of tissue. The lower two data points for naive mice on day 2 were from a preliminary experiment. (*G*) Virus titers in nasal turbinate on days 2 and 5 were determined as in *F* and plotted as TCID50 per sample. (*H*) RNA was isolated from lung homogenates on days 2 and 5. CoV-2 N and S subgenomic RNAs were determined by ddPCR and plotted as copies per microgram of RNA. (*I*) RNA was isolated from nasal turbinate homogenates as described in *H*. Significance (*P* < 0.004) was determined by combining the values for N mRNAs or S mRNAs on day 2 from both control groups and comparing that to the combined values from rMVA vaccinated mice using Student’s *t* test. (*J*) Cytokine and chemokine RNAs in day 2 lung homogenates were determined by ddPCR. Values for control and naive mice were plotted together, as were those for rMVAs that received homologous boosts. X1 refers to sera collected 3 wk after prime; X2 refers to sera collected 2 wk after homologous boost; RBD Pro in red refers to heterologous boost with RBD protein.

Transgenic mice that express hACE2 regulated by the cytokeratin 18 (K18) gene promoter (K18-hACE2) (43) are highly susceptible to intranasal (IN) CoV-2 infection (44). High levels of virus are present in the lungs within a few days, and severe weight loss occurs by day 5 or 6, with animals becoming moribund. A total of 36 hACE2 transgenic mice, of which 12 were controls and 24 were vaccinated with rMVAs, were used in the experiment depicted in Fig. 5. The controls were subdivided into unvaccinated (naive) and those vaccinated with parental MVA lacking CoV-2 sequences. The latter were included to evaluate potential nonspecific protective effects due to innate immune responses to the MVA vector. The 24 remaining mice were divided into groups of six that received IM inoculation with *2P* or *Tri* and boosted 3 wk later with the homologous rMVA or with RBD protein in adjuvant as depicted in Fig. 5*A*. In this way, four different vaccine modalities were evaluated simultaneously. Binding antibody to S was detected 3 wk after the *2P* and *Tri* primes and was boosted up to 10-fold with homologous or protein boosts (Fig. 5*B*). Isotype analysis indicated that the binding antibody was IgG2c > IgG2b > IgG1> IgG3 and no detectable IgA (Table 1), indicating a strong Th1 response in the hACE2 mice receiving *2P* or *Tri* and homologous or heterologous boosts. In contrast to binding antibody, CoV-2 neutralizing antibody was boosted by RBD protein but not appreciably by the rMVAs (Fig. 5*C*). Further experiments may be needed to optimize the time interval between rMVA prime and boost inoculations as immune responses to the vectors are elicited (*SI Appendix*, Fig. S4).

At 2 wk after boosts, hACE2 mice were infected IN with 10^5^ TCID50 (defined as the median tissue culture infectious dose) of CoV-2. The naive and parental MVA immunized mice lost weight by day 5 and died or met the predetermined criteria for euthanasia on day 6 (Fig. 5*D*). The similarity between the two controls indicated that nonspecific innate immune responses due to the parental MVA were not protective. In contrast, each of the four groups of rMVA-vaccinated mice, whether boosted with rMVA or RBD protein, lost no weight and appeared healthy throughout the experiment. The surviving mice were rechallenged with CoV-2 after 2 wk and again showed no weight loss, whereas additional naive mice succumbed to the virus infection by day 6 (Fig. 5*E*).

Lung homogenates of naive and parental MVA immunized control mice had CoV-2 titers of >10^7^ TCID50 per gram of tissue on day 2, which dropped slightly on day 5, whereas no infectious virus was found in any of the 16 rMVA-vaccinated mice analyzed at either time regardless of the prime or boost (Fig. 5*F*). The virus titers in the nasal turbinates of control mice peaked at day 2 but were still elevated at day 5 (Fig. 5*G*). In contrast, only one rMVA-vaccinated mouse had a low level of virus in the turbinates that was more than two logs lower than controls on day 2, and none had detectable virus on day 5.

Subgenomic N and S mRNAs were analyzed by digital droplet PCR (ddPCR) using specific primers to distinguish newly synthesized mRNA from input viral RNA. High levels of N and S mRNA were found in lungs of both control groups on days 2 and 5 (Fig. 5*H*). In contrast, subgenomic mRNAs were undetectable or reduced by four or more logs in the lungs of rMVA-vaccinated animals (Fig. 5*H*). N and S mRNAs were also detected in the nasal turbinates of control mice on both days but had decreased about a log between the two times (Fig. 5*I*). In the nasal turbinates of rMVA-vaccinated mice, N and S mRNAs were reduced by more than two logs on day 2 compared to controls and were detectable in only one of eight mice on day 5. Statistical significance (*P* < 0.004) was determined by combining the values for N mRNAs or S mRNAs on day 2 from both control groups and comparing that to the combined values from all rMVA-vaccinated mice. Thus, the rMVA-vaccinated mice exhibited a high degree of protection against CoV-2 regardless of whether they were primed with *2P* or *Tri* and boosted with the homologous rMVA or RBD protein.

Cytokine and chemokine RNAs in lung homogenates on day 2 after challenge were quantified by ddPCR. The values for the two controls (naive and MVA) were grouped together, as were the rMVA prime−boosts (*2P* and *Tri*), in Fig. 5*J*. The levels of IFNβ, IFNγ and IFNλ were approximately three logs lower in the rMVA-vaccinated mice than in the controls. TNFα, IL6, CXCL 10, CXCL 11, CCL 2, and CCL 5 were also lower in the rMVA-vaccinated mice than in the controls.

### A Single Vaccination with an rMVA Protects Transgenic Mice from CoV-2.

**Fig. 6.**
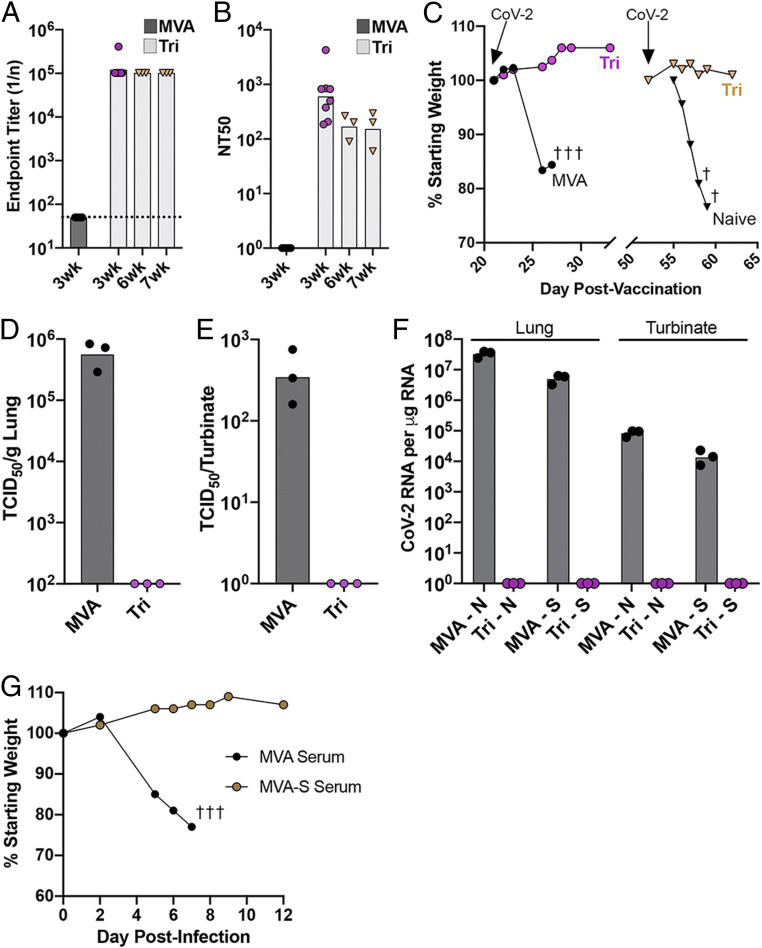
Challenge of transgenic mice after a single vaccination and after passive transfer of immune sera. (*A*–*F*) K18-hACE2 mice (female, 7 wk old) were vaccinated IM with 2 × 10^7^ PFU of MVA (*n* = 5) or rMVA *Tri* (*n* = 8), and five mice of each group were challenged 3 wk later with 10^5^ TCID50 of CoV-2 IN. (*A*) S-binding antibody prior to challenge. (*B*) Neutralizing antibody prior to challenge. (*C*) Weights of mice after challenge. Symbol † indicates number of mice that died or were euthanized on a specific day. (*D*) Lung virus titers. (*E*) Nasal turbinate virus titers. (*F*) N and S subgenomic RNA copies per microgram of RNA in lung and nasal turbinates. (*G*) Passive serum transfer. K18-hACE2 mice were injected intraperitoneally with 0.4 mL of pooled serum from BALB/c mice vaccinated with parental MVA (*n* = 3) or with MVA-S (*n* = 4) and challenged with 10^5^ TCID50 of CoV-2 IN. Mice were weighed on the indicated days, and values are plotted as percent of starting weight of each mouse. Symbol † indicates number of mice that died or were euthanized on day 7.

The high levels of neutralizing antibody after priming with *2P* and *Tri* led us to investigate whether a single rMVA vaccination would protect hACE2 mice against an intranasal challenge with CoV-2. The hACE2 mice were vaccinated with parental MVA or *Tri* and challenged 21 d or 51 d later. Between the two challenges, the binding antibody was undiminished, but the neutralizing antibody fell by about one log (Fig. 6 *A* and *B*). Following IN administration of CoV-2, mice that received the parental MVA suffered severe weight loss and died or met the criteria for euthanasia, whereas the mice that received *Tri* lost no weight and remained healthy after the 21- and 51-d challenges (Fig. 6*C*). The lungs and turbinates were analyzed 5 d after the 21-d challenge. No infectious CoV-2 or subgenomic N or S mRNA was detected (Fig. 6 *D*–*F*).

### Protection of hACE2 Mice by Passive Transfer of Serum.

A passive transfer experiment was carried out to determine whether antibody induced by vaccination with rMVA S vectors is sufficient to protect against lethal infection with CoV-2. Sera were pooled from mice that had been vaccinated by priming and boosting with parental MVA or rMVA expressing WT S. Aliquots were injected into the peritoneum of hACE2 mice, which were challenged with CoV-2 ∼24 h later. A few hours before the challenge, the mice were bled, and pseudotype neutralizing titers of 160 NT50 were found for each of the mice that received the immune serum. Mice that received immune serum showed no signs of weight loss or ill health following inoculation of CoV-2 (Fig. 6*G*).

## Discussion

In an endeavor to optimize the synthesis and immunogenicity of S, the major target of CoV-2 neutralizing antibodies, we constructed a panel of rMVAs that expressed unmodified and modified versions of S. A common feature of all the rMVAs was good surface expression of the RBD as shown by interaction with an anti-RBD mAb and soluble hACE2. Each of the rMVAs induced neutralizing antibodies (NT50 of 10^3^ or higher) that compared favorably with that achieved by mRNA immunizations in mice (11) and in a phase 1 clinical trial (45) using the same lentivirus pseudovirus CoV-2 neutralization protocol and reagents. Neutralizing antibody was detected in mice at 1 wk after vaccination, peaked at 3 wk, and persisted for at least 7 wk. In some experiments, rMVA *Tri* containing S with proline stabilizing mutations, inactivation of the furin cleavage site, and deletion of the ERRS elicited slightly higher neutralizing antibody than other rMVAs after a single immunization, but larger numbers of mice would be needed to confidently assess superiority.

Isotype analysis indicated that the rMVAs induced a well-balanced predominantly Th1-type response to S with IgG2a or IgG2c (depending on the mouse strain) > IgG2b > IgG1 > IgG3, which is the usual order following a viral infection and by IFNγ stimulation (41, 46, 47). IgG2a, IgG2b, and IgG2c have similar functions and are able to fix complement and also activate Fc receptors to promote virus clearance, whereas IgG1 may limit inflammation (41). Lower levels of binding and neutralizing antibodies were detected following priming and boosting with purified soluble RBD protein in QS-21 adjuvant. IgG1 was predominant when mice were immunized with the adjuvanted RBD protein, even though more balanced IgG1 and IgG2 responses have been reported using other immunogens with QS-21 (48). Nevertheless, the predominance of IgG2a and IgG2c was maintained and the levels increased when RBD protein was used as the boost for rMVAs, suggesting that the protein stimulated an anamnestic response. The trade-off of boosting with RBD protein, however, was a lower CD8+IFNγ+ T cell response.

Vaccine protection studies were carried out with transgenic K18-hACE2 mice, which exhibit fatal respiratory infection following IN administration of CoV-2 (44, 49–51). Two rMVAs were selected for immunization: one (*2P*) expressed full-length S with the two proline substitutions like that used in other vaccines studies, and the other (*Tri*) had two additional modifications. Mice were primed with the rMVAs and boosted with the homologous rMVA or adjuvanted RBD protein. Each of the four vaccination protocols successfully prevented weight loss and morbidity. No infectious CoV-2 was recovered from the lungs, and cytokine and chemokine levels were severely reduced. Nevertheless, low amounts of subgenomic N and S mRNAs were detected in the nasal turbinates of some mice at 2 d after challenge, indicating that abortive CoV-2 replication occurred. Further experiments demonstrated that a single vaccination with an rMVA protected transgenic mice when they were challenged with CoV-2 at 3 wk or 7 wk, even though the neutralizing antibody titer had dropped significantly at the later time.

Although the rMVAs stimulated antibody and CD8+ T cells, passive transfer of serum from vaccinated mice suggested that antibodies were sufficient for protection of transgenic mice against fatal CoV-2 infection. In addition, mice that were primed with rMVAs and boosted with RBD protein protected as well as mice that were boosted with rMVAs, even though the protein boost did not stimulate increased CD8+ T cells. Nevertheless, these experiments do not rule out a contribution of CD8+ T cells to immunity.

Advantages of MVA as a vector includes safety in children and immunocompromised individuals as well as stability for at least 24 mo frozen and 12 mo at 2 °C to 8 °C (52). However, a pertinent question is whether elderly recipients who received the smallpox vaccine decades ago might make lower immune responses to an rMVA because of persistent anti-VACV neutralizing antibodies (53, 54). This question was investigated previously in the context of rMVA HIV vaccine studies. Macaques that had been vaccinated with the standard smallpox vaccine made undiminished anti-simian immunodeficiency virus (SIV) ENV and GAG antibodies and controlled an SIV challenge when vaccinated with rMVAs 17 mo later, although CD8+T cells were reduced (55). Another macaque study showed that SIV antibody increased with a second and a third boost of rMVA (56). These nonhuman primate studies suggest that residual immunity from smallpox vaccination may not be a significant problem for an MVA-based COVID-19 vaccine.

In the present study, we evaluated rMVA CoV-2 vaccines administered IM. The absence of detectable anti-S IgA in serum was in accord with the result of Hassan et al. (13), who found IgA after IN but not IM vaccination with an adenovirus CoV-2 S vaccine. MVA can be administered orally, IN, and by aerosol, and the IN route has been shown to stimulate bronchus-associated lymphoid tissue and serum IgA (57–59). Even though IM administration of the rMVA CoV-2 S vaccines greatly reduced and rapidly eliminated virus replication in the nasal turbinates, IN administration might have a greater ability to reduce CoV-2 spread. Nevertheless, complete prevention of viral replication may prove difficult, as low amounts of viral RNA were still detected in the turbinates when an adenovirus vector expressing CoV-2 S was inoculated IN (13).

We showed that RBD and full-length S protein can boost antibody responses as well as or slightly better than an rMVA boost. There is precedent for other heterologous prime−boost systems in which MVA is teamed up with nucleic acids and other virus vectors (60–62), providing another potential vaccine strategy to combat COVID-19.

After submission of our manuscript for review, two related studies were posted online in advance of publication (19) and as a preprint (63). In both studies, rMVA vectors that expressed unmodified full-length CoV-2 S induced neutralizing antibodies and protected mice that expressed hACE-2 from infection with CoV-2.

### Limitations of Study.

K18-hACE2 transgenic mice are imperfect models of human susceptibility to CoV-2, and the virus inoculum, which caused severe morbidity and death within 5 d to 6 d, is likely higher than occurs during human transmission. The lack of signs of morbidity, failure to detect infectious CoV-2 in the lungs, and reduced levels of cytokines and chemokines in the lungs of vaccinated mice are consistent with the prevention of pathology, although the latter was not evaluated by pulmonary function tests or histological examination.

## Materials and Methods

Detailed procedures and sources of reagents are described in *SI Appendix*.

### Mice.

The 5- to 6-wk-old female BALB/cAnNTac and C57BL/6ANTac mice were obtained from Taconic Biosciences, and B6.Cg-Tg(K18-ACE2)2Prlmn/J mice were obtained from Jackson Laboratories.

### Construction of Recombinant Viruses.

DNA encoding the CoV-2 S protein (QHU36824.1), with a C-terminal 3×FLAG tag and modified by silent mutations for enhanced expression and stability in MVA, was chemically synthesized. Another construct (*Tri*) with the proline substitutions (K986P/V987P), furin recognition site substitutions (aa 682 to 685 RRAR to GSAS), and C-terminal 19-aa deletion of ERRS was also synthesized. Constructs with these single mutations were generated by site-directed mutagenesis. The rMVAs were produced by homologous recombination, clonally purified by repeated plaquing, and physically purified by sedimentation through sucrose.

### Detection of S Protein by Flow Cytometry.

For intracellular staining, cells were fixed and permeabilized and incubated with anti-CoV-2 Spike RBD mAb followed by APC-conjugated goat anti-mouse IgG antibody. For surface detection, the cells were stained directly using the same primary and secondary antibodies. The binding of hACE2 to surface-expressed S protein on infected HeLa cells was detected by incubating with biotinylated human ACE2 protein followed by Alexa Fluor 647-conjugated anti-hACE2 antibody. The stained cells were acquired on a FACSCalibur cytometer using Cell Quest software and analyzed with FlowJo software.

### Detection of S-Binding Antibodies by ELISA.

CoV-2 S protein produced in HEK293 cells was adsorbed to wells of a 96-well flat-bottom plate and incubated with serum. After adsorption, the wells were washed, blocked, washed again, and incubated with dilutions of serum. HRP-conjugated goat anti-mouse IgG (H+L) was added to each well. Spectrophotometric measurements were made at A_450_ and A_650_ using a plate reader. Final end point titers (1/n) for each sample were determined as fourfold above the average optical density (OD) of those wells not containing primary antibody (OD 0.03 to 0.04).

### Stimulation and Staining of Lymphocytes.

Splenocytes were mixed with individual peptide pools in 96-well plates and incubated at 37 °C for 1 h, after which Brefeldin A was added and incubation continued for 4 h to 5 h. Surface staining was performed with anti-mouse CD3-FITC, anti-mouse CD4-PE, and anti-CD8-PerCP-Cy5.5 for 1 h. Cells were then fixed and permeabilized with Cytofix/Cytoperm solution and stained with IFNγ-APC and fixed with 2% paraformaldehyde. Approximately 100,000 events were acquired on a FACSCalibur cytometer using Cell Quest software and analyzed with FlowJo software.

### Pseudovirus Neutralization Assay.

The CoV-2 lentivirus pseudotype assay was carried out as described by Corbett et al. (11) using cells and plasmids obtained from the National Institute of Allergy and Infectious Diseases (NIAID) Vaccine Research Center. Luciferase units (relative light units) were read on a luminometer, and the NT50 was calculated using Prism to plot dose–response curves, normalized using the average of the no virus wells as 100% neutralization, and the average of the no serum wells as 0%.

### MVA Neutralization Assay.

A semiautomated flow cytometric assay was carried out as previously described (64) except for substitution of MVA expressing GFP for the WR strain of VACV. The dilution of mouse serum that reduced the percentage of GFP-expressing cells by 50% was determined by nonlinear regression using Prism.

### CoV-2 Challenge Virus.

CoV-2 USA-WA1/2019 was obtained from BEI Resources (Ref# NR-52281) and propagated in a biosafety level-3 (BSL-3) laboratory using Vero E6 cells by Bernard Lafont of the NIAID SARS Virology Core laboratory. The TCID50 of the clarified culture medium was determined on Vero E6 cells after staining with crystal violet, and was scored by the Reed−Muench method.

### Vaccination and Challenge Experiments.

The rMVAs (1 × 10^7^ PFU in 50 µL) were injected IM into each hind leg of the mouse. After 3 wk, the mice received a second injection of the homologous rMVA or an injection of 10 µg of baculovirus RBD protein with QS-21 adjuvant in the left hind leg. Mice were lightly sedated with isoflurane and inoculated IN with 50 µL of SARS-CoV-2 (10^5^ TCID50). After infection, morbidity/mortality status and weights were assessed and recorded daily for 14 d.

### Determination of CoV-2 in Lungs and Nasal Turbinates.

Lungs and nasal turbinates were homogenized, cleared of debris by centrifugation, and titrated in quadruplicate on Vero E6 cells using 10-fold serial dilutions in 96-well microtiter plates. After 72 h to 96 h, the plates were stained with crystal violet and scored using the Reed−Muench method to determine TCID50.

### Determination of CoV-2 RNA in Lungs and Nasal Turbinates.

RNA was extracted from homogenates of lungs and turbinates using TRIzol, contaminating DNA was removed, and RNA was reverse transcribed. CoV-2 S and N transcripts and 18s ribosomal RNA were quantified by ddPCR with specific primers using an automated droplet generator and droplet reader.

### Passive Serum Transfer.

Pooled serum for passive transfer was obtained from BALB/c mice that had been primed and boosted with rMVA S (*WT*) or parental MVA. Naive K18-hACE2 mice received 0.4 mL of rMVA S or control MVA serum. The following day, mice were bled to determine levels of SARS-CoV-2 binding and neutralizing antibody. Approximately 4 h later, the mice were challenged IN with 10^5^ TCID50 of CoV-2. Mice were observed and weighed over the next 2 wk.

### Safety and Ethics.

All experiments and procedures involving mice were approved under protocol LVD29E by the NIAID Animal Care and Use Committee according to standards set forth in the NIH guidelines, Animal Welfare Act, and US Federal Law. Euthanasia was carried out using carbon dioxide inhalation in accordance with the *American Veterinary Medical Association guidelines for euthanasia of animals* (2013 Report of the AVMA Panel of Euthanasia) (65). Experiments with SARS-CoV-2 were carried out under BSL-3 containment.

## Supplementary Material

Supplementary File

## Data Availability

All study data are included in the article and *SI Appendix*.
