# Supplementary material for: One or two injections of MVA-vectored vaccine shields hACE2 transgenic mice from SARS-CoV-2 upper and lower respiratory tract infection
Source: Proc Natl Acad Sci U S A. 2021 Mar 9;118(12):e2026785118. doi: 10.1073/pnas.2026785118 (PMC8000198; doi:10.1073/pnas.2026785118)
Supplement: Supplementary File [file pnas.2026785118.sapp.pdf]

PNAS
www.pnas.org

Supplementary Information for

One or Two Injections of MVA-Vectored Vaccine Shields hACE2 Transgenic Mice From SARS-CoV-2 Upper and Lower Respiratory Tract Infection

Ruikang Liu, Jeffrey L. Americo, Catherine A. Cotter, Patricia L. Earl, Noam Erez, Chen Peng and Bernard Moss

Corresponding author: Bernard Moss
Email: bmoss@nih.gov

This PDF file includes:

Supplementary text
Figures S1 to S7
SI References

Supplementory Information Text
Materials and Methods

Mice. Five-to six-week-old female BALB/cAnNTac and C57BL/6ANTac were obtained from Taconic Biosciences and B6.Cg-Tg(K18-ACE2)2Prlmn/J from Jackson Laboratories. Mice were separated into groups of 2-5 animals in small, ventilated microisolator cages in an ABSL-2 facility and used after 1-5 additional weeks.

Construction of Recombinant Viruses. DNA encoding the CoV-2 S protein (QHU36824.1), with a C-terminal 3xFLAG tag and modified by removing four VACV early transcription termination signals (TTTTTNT) and runs of four or more consecutive Gs or Cs, was chemically synthesized (Thermo Fisher). Another construct (*Tri*) with the proline substitutions (K986P/V987P), furin recognition site substitutions (aa 682–685 RRAR to GSAS), and C-terminal 19 aa deletion of ERRS was also synthesized. Constructs with these individual mutations were generated using Q5 Site-Directed Mutagenesis Kit (New England Biolabs). A 2-step PCR protocol using the Q5 mutagenesis kit was used to join nucleotides 1-117, 955-1782 and 3586-3819 to form the ORF for *RBD*. The DNAs were inserted into the pLW44 transfer vector (1) at the *Xma*I and *Sal*I sites, which placed the ORF under the control of the VACV modified H5 early late promoter and adjacent to the separate gene encoding enhanced GFP regulated by the VACV P11 late promoter.

To produce rMVAs, linearized plasmids were transfected into cells infected with MVA allowing recombination into the existing deletion III site in the MVA genome (1). The rMVAs were clonally purified by four successive rounds of fluorescent plaque isolation, propagated in CEF, and purified by sedimentation twice through a 36% sucrose cushion. The genetic purities of the recombinant viruses were confirmed by PCR amplification and sequencing of the modified region. Titers of MVAs were determined in CEF by staining plaques with anti-VACV rabbit antibodies (1).

Western Blotting. HeLa cells were infected with 5 PFU per cell of rMVAs for 18 h, washed once with phosphate buffered saline (PBS), then lysed in LDS sample buffer with reducing agent (Thermo Fisher). The lysates were dispersed in a sonicator for four 30 s periods; the proteins were resolved on 4 to 12% NuPAGE Bis-Tris gels (Thermo Fisher) and transferred to a nitrocellulose membrane with an iBlot2 system (Thermo Fisher). The membrane was blocked with 5% nonfat milk in Tris-buffered saline (TBS) for 1 h, washed with TBS with 0.1% Tween 20 (TBST), and then incubated at 4°C overnight with rabbit anti-CoV-2 RBD polyclonal antibody (Cat# 40592-T62, Sino Biological) or anti-FLAG M2 peroxidase antibody (Cat# A8592, MilliporeSigma) in 5% nonfat milk in TBST. The membrane that had been incubated with anti-RBD antibody was then incubated for 1 h with secondary antibody conjugated to horseradish peroxidase (Jackson ImmunoResearch). After washing, the membrane bound proteins were detected with SuperSignal West Dura substrate (Thermo Fisher).

Detection of S Protein by Flow Cytometry. HeLa cells were infected with 5 PFU per cell of rMVAs. After 24 h, the infected cells were stained for intracellular and surface S in parallel. For intracellular staining, cells were fixed with Cytofix/Cytoperm (BD Biosciences), permeabilized with Perm/Wash Buffer (BD Biosciences), and incubated with anti-CoV-2 Spike RBD mAb (SARS2-02) (2) followed by APC-conjugated goat anti-mouse IgG antibody (Cat# 405308,

BioLegend). For surface detection, the cells were stained directly using the same primary and secondary antibodies. The binding of hACE2 to surface expressed S protein on infected HeLa cells was detected by incubating with 100 ng/10^6 cells of biotinylated human ACE2 protein (Cat# 10108-H08H-B, Sino Biological) followed by Alexa Fluor 647-conjugated anti-hACE2 antibody (Cat# FAB9332R, R&D Systems). The stained cells were acquired on a FACSCalibur cytometer using Cell Quest software and analyzed with FlowJo (BD Biosciences).

Detection of S-Binding Antibodies by ELISA. CoV-2 S protein produced in HEK293 cells was obtained from the NIAID Vaccine Research Center or Sino Biological and diluted in cold PBS to a concentration of 1 µg/ml. Diluted S protein (100 µl) was added to each well of a MaxiSorp 96-well flat-bottom plate (Thermo Fisher). After incubation for 16-18 h at 4°C, the wells were washed 3 times with 250 µl of PBS + 0.05% Tween 20 (PBS-T, Accurate Chemical) and plates were blocked with 200 µl PBS-T + 5% Nonfat Dry Milk for 2 h at room temperature. During the blocking phase, a series of eight 4-fold dilutions of each mouse serum sample was prepared in blocking buffer. After blocking, plates were washed 3 times with 250 µl of PBS-T and 100 µl of each 4-fold dilution of serum was added to the appropriate well(s) and incubated for 1 h at room temperature. After incubation with serum, plates were washed 3 times with 250 µl of PBS-T. HRP-conjugated goat anti-mouse IgG (H+L) (Thermo Fisher) was diluted 1:4000 in blocking buffer and 100 µl of the secondary antibody was added to each well for 1 h at room temperature. For detection of antibody isotypes, peroxidase-conjugated isotype-specific antibodies were used (Thermo Fisher). Plates were washed 3 times with 250 µl of PBS-T and then 100 µl of KPL SureBlue TMB 1-component microwell peroxidase substrate (SeraCare) was added to each well. The chemiluminescence reaction was stopped after 10 min by addition of 100 µl of 1N sulfuric acid. Spectrophotometric measurements were made at A_{450} and A_{650} using a SpectraMax Plus 384 plate reader with SoftMax Pro analysis software (Molecular Devices). Final endpoint titers (1/n) for each sample were determined as 4-fold above the average OD of those wells not containing primary antibody (OD 0.03-0.04).

Stimulation and Staining of Lymphocytes. Splenocytes from individual mice or pooled from 3-5 mice were suspended at 1.5×10^7 cells/ml in RPMI (Quality Biological) supplemented with 10% heat-inactivated FBS, 10 U/ml penicillin, 10 µg/ml streptomycin, 2 mM L-glutamine, and 2 mM HEPES as previously described (3). Splenocytes (100 µl) were mixed with 100 µl of individual peptide pools in 96-well plates and incubated at 37°C for 1 h after which brefeldin A (Sigma Aldrich) was added and incubation continued for 4-5 h. Staining of cells was performed at 4°C. Fc receptors were blocked with anti-CD16/32 (Clone 2.4G2, a gift from Jack Bennink, NIAID) for 30 min. Surface staining was performed with anti-mouse CD3-FITC (Clone17A2; BioLegend), anti-mouse CD4-PE (Clone H129.19; BD Biosciences), and anti-CD8-PerCP-Cy5.5 (BD Biosciences) for 1 h. Cells were then fixed and permeabilized with Cytofix/Cytoperm solution and stained with IFNγ-APC (BD Biosciences) for 1 h. Cells were washed with PBS and suspended in PBS containing 2% paraformaldehyde. Approximately 100,000 events were acquired on a FACSCalibur cytometer using Cell Quest software and analyzed with FlowJo (BD Biosciences).

A peptide array was obtained from BEI Resources (catalog # NR-52402; SARS-Related Coronavirus 2 Spike (S) Glycoprotein). Each peptide was dissolved in DMSO at 10 µg/ml. A total of 18 peptide pools were prepared, containing 3-11 peptides per pool. For splenocyte

stimulation, the final concentration of each peptide was 2 µg/ml. Peptides in the 2 positive pools were: Pool #4 (BEI peptides 32-41); Pool #7 (BEI peptides 61, 64, 77).

Pseudovirus Neutralization Assay. The CoV-2 lentivirus pseudotype assay was carried out as described by Corbett et al. (4) using cells and plasmids obtained from the NIAID Vaccine Research Center. To determine neutralization titers, serum samples were heat inactivated for 30 min at 56°C and clarified by high speed microcentrifugation. The day before titration, 5,000 293T-hACE2.MF cells were seeded per well in 96-well white walled clear bottom tissue culture plates (Corning) in DMEM supplemented with 10% heat inactivated FBS, 2 mM L-glutamine, 100 U/ml penicillin, 100 µg/ml streptomycin with 3 µg/ml of puromycin. For each serum sample, duplicate 4-fold dilution series were prepared in 96-well U-bottom plates (Corning) in DMEM supplemented with 5% heat inactivated FBS with the starting dilution being 1:20 in a final volume of 45 µl per well. The pseudovirus was thawed at 37°C and 45 µl of a dilution previously shown to exhibit a 1000-fold difference in luciferase between uninfected and infected cells was added to all wells except for controls. After 45 min at 37°C, the medium was aspirated and 50 µl sample-virus mixture was added to each well and incubated for 2 h at 37°C. DMEM (150 µl) supplemented with 5% heat inactivated FBS was added per well and plates were incubated for 72 h at 37°C. Medium was removed from the wells and the cells were lysed with 25 µl per well of 1X cell lysis reagent (Promega), shaken at 400 rpm for 15 min at room temperature. Luciferase reagent (50 µl, Promega) was added per well and 90 s later relative luciferase units (RLU) were read on the luminometer (EnSight, Perkin Elmer, 570 nm wavelength, 0.1 mm distance, 0.3 s read). NT50 were calculated using Prism (GraphPad Software) to plot dose-response curves, normalized using the average of the no virus wells as 100% neutralization, and the average of the no serum wells as 0%.

MVA Neutralization Assay. A semi-automated flow cytometric assay was carried out as previously described (5) except for substitution of MVA expressing GFP for the WR strain of VACV. Briefly, ten 2-fold serial dilutions of heat-inactivated serum from vaccinated mice were prepared in a 96-well plate and 6.25×10^3 PFU of MVA-GFP was added to each well and incubated at 37°C for 1 h. Approximately 10^5 HeLa suspension cells were added to each well in the presence of 44 µg/ml of cytosine arabinoside. After 18 h at 37°C, the cells were fixed in 2% paraformaldehyde and acquired with a FACSCalibur cytometer using Cell Quest software and analyzed with FlowJo. The dilution of mouse serum that reduced the percentage of GFP-expressing cells by 50% (IC_{50}) was determined by nonlinear regression using Prism.

CoV-2 Challenge Virus. CoV-2 USA-WA1/2019 was obtained from BEI resources (Ref# NR-52281) and propagated in a BSL-3 laboratory using Vero E6 cells cultured in DMEM+Glutamax supplemented with 2% heat-inactivated FBS and penicillin, streptomycin, and fungizone by Bernard Lafont of the NIAID SARS Virology Core laboratory. The TCID50 of the clarified culture medium was determined on Vero E6 cells after staining with crystal violet and scored by the Reed-Muench method.

Vaccination and Challenge Experiments. Prior to vaccination, the virus was thawed, sonicated twice for 30 s on ice and diluted to 2×10^8 PFU/ml in PBS supplemented with 0.05% bovine serum albumin. In an ABSL-2 laboratory, 50 µl of diluted virus was injected IM into each hind leg of the animal for a total dose of 2×10^7 PFU. Unless otherwise stated, baculovirus RBD

protein provided by Eugene Valkov (NCI) was diluted to 0.2 mg/ml in PBS containing 0.3 mg/ml of QS-21 adjuvant (Desert King International, San Diego, CA) and 10 µg of RBD was injected IM into the left hind leg. All mice scheduled to be infected with SARS-CoV-2 were transferred to an ABSL-3 laboratory a few days prior to virus challenge. The challenge stock of SARS-CoV-2 USA-WA1/2019 was diluted to 2 x 10^6 TCID50/ml in PBS. Mice were lightly sedated with isoflurane and inoculated IN with 50 µl of SARS-CoV-2. After infection, morbidity/mortality status and weights were assessed and recorded daily for 14 days by the NIAID Comparative Medical Branch.

Determination of CoV-2 in Lungs and Nasal Turbinates. At 2- and 5-days post-infection with CoV-2, lung and nasal turbinates were removed and placed in 1.5-2 ml of ice-cold Dulbecco's PBS and weights of lungs were recorded. Tissues were homogenized for three 25 s intervals in ice water using a GLH-1 grinder equipped with a disposable probe and aerosol proof cap (Omni International). Homogenates were cleared of debris by centrifugation at 4,000 xg for 10 min and the supernatants were transferred to sterile tubes and stored at -80°C. Clarified homogenates were thawed and titrated in quadruplicate on Vero E6 cells using 10-fold serial dilutions in 96-well microtiter plates. After 72-96 h, the plates were stained with crystal violet and scored using the Reed-Muench method to determine TCID50.

Determination of CoV-2 RNA in Lungs and Nasal Turbinates. Immediately after homogenization of lungs and turbinates, 0.125 ml was transferred to sterile tubes, 0.9 ml Trizol (Thermo Fisher) was added and the mixture frozen. After thawing, RNA was extracted using the Trizol Plus RNA Purification Kit with Phasemaker tubes (Thermo Fisher) following the manufacturer's instructions. Contaminating DNA was removed from the eluted RNA using the Turbo DNA-free kit (Thermo Fisher) and RNA was reverse-transcribed using the iScript cDNA synthesis kit (Bio-Rad, Hercules, CA). CoV-2 S and N transcripts and 18s rRNA were quantified by ddPCR with specific primers (CoV-2 RNA Leader, Forward – CGA TCT CTT GTA GAT CTG TTC TCT AAA C; CoV-2 S, Reverse – TCT TAG TAC CAT TGG TCC CAG AGA; CoV-2 N, Reverse - GGT CTT CCT TGC CAT GTT GAG T; 18S, Forward - GGC CCT GTA ATT GGA ATG AGT C; 18S, Reverse - CCA AGA TCC AAC TAC GAG CTT) using an automated droplet generator and QX200 Droplet Reader (Bio-Rad). The values for CoV-2 S transcripts were normalized using the 18s RNA in the same sample.

Passive Serum Transfer. Serum for passive transfer was obtained from 20 BALB/c mice that were inoculated IM with rMVA S (*WT*) and 10 BALB/c mice with parental MVA at 0 and 3 weeks. Two weeks after the boosts, the MVA S and control MVA sera were pooled separately. Four naive K18-hACE2 mice each received 0.4 ml of MVA S serum and three received 0.4 ml of the control MVA serum. The following day, mice were bled to determine levels of SARS-CoV-2 binding and neutralizing antibody. Approximately 4 h later, the mice were challenged IN with 10^5 $TCID_{50}$ of CoV-2. Mice were observed and weighed over the next two weeks.

Safety and Ethics. All experiments and procedures involving mice were approved under protocol LVD29E by the NIAID Animal Care and Use Committee according to standards set forth in the NIH guidelines, Animal Welfare Act, and US Federal Law. Euthanasia was carried out using carbon dioxide inhalation in accordance with the American Veterinary Medical

Association Guidelines for Euthanasia of Animals (2013 Report of the AVMA Panel of Euthanasia). Experiments with SARS-CoV-2 were carried out under BSL-3 containment.

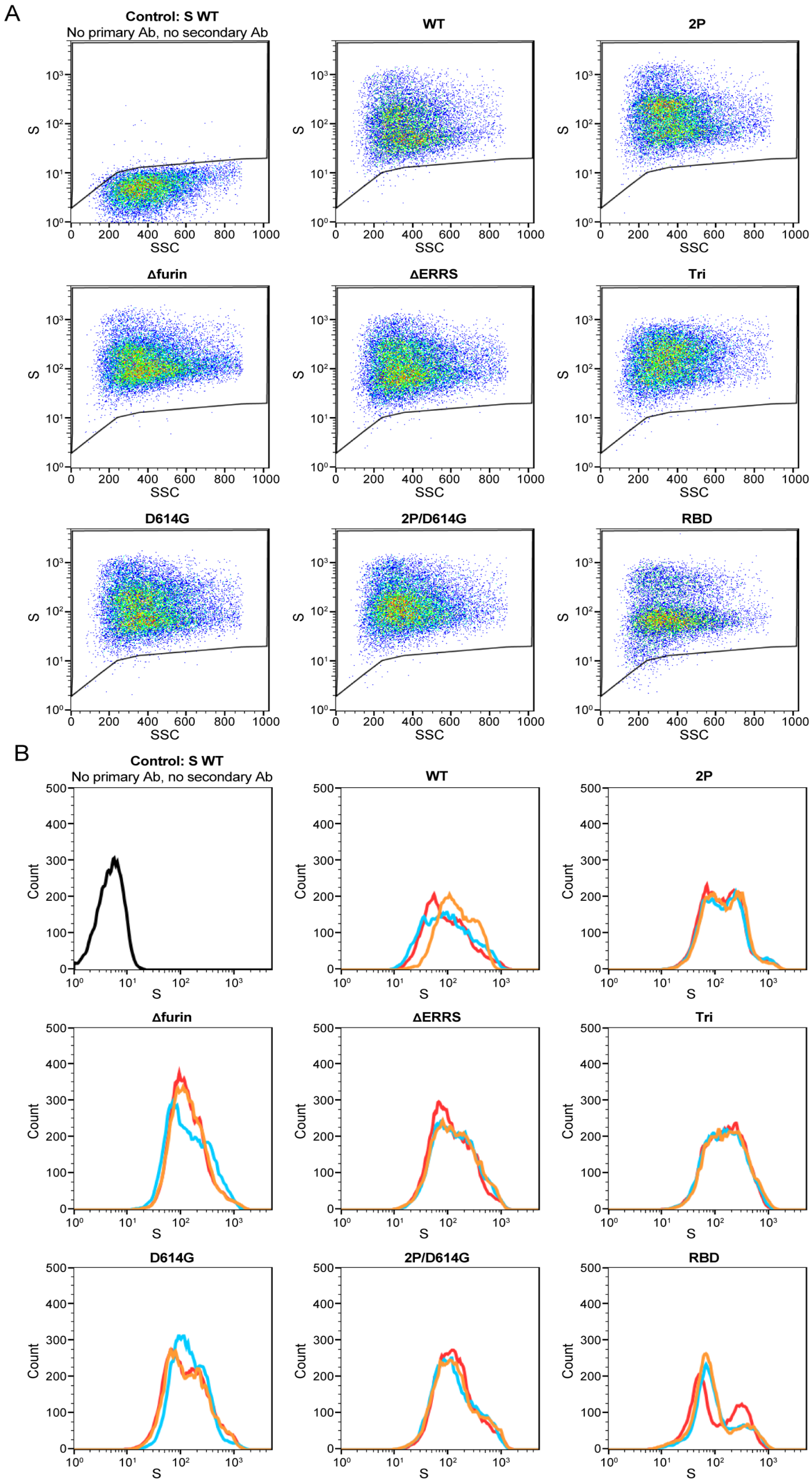

Fig. S1. Scatter plots and histograms showing anti-RBD antibody binding to rMVA-infected permeabilized HeLa cells related to Fig. 2C. (A) Scatter plots. (B) Histograms.

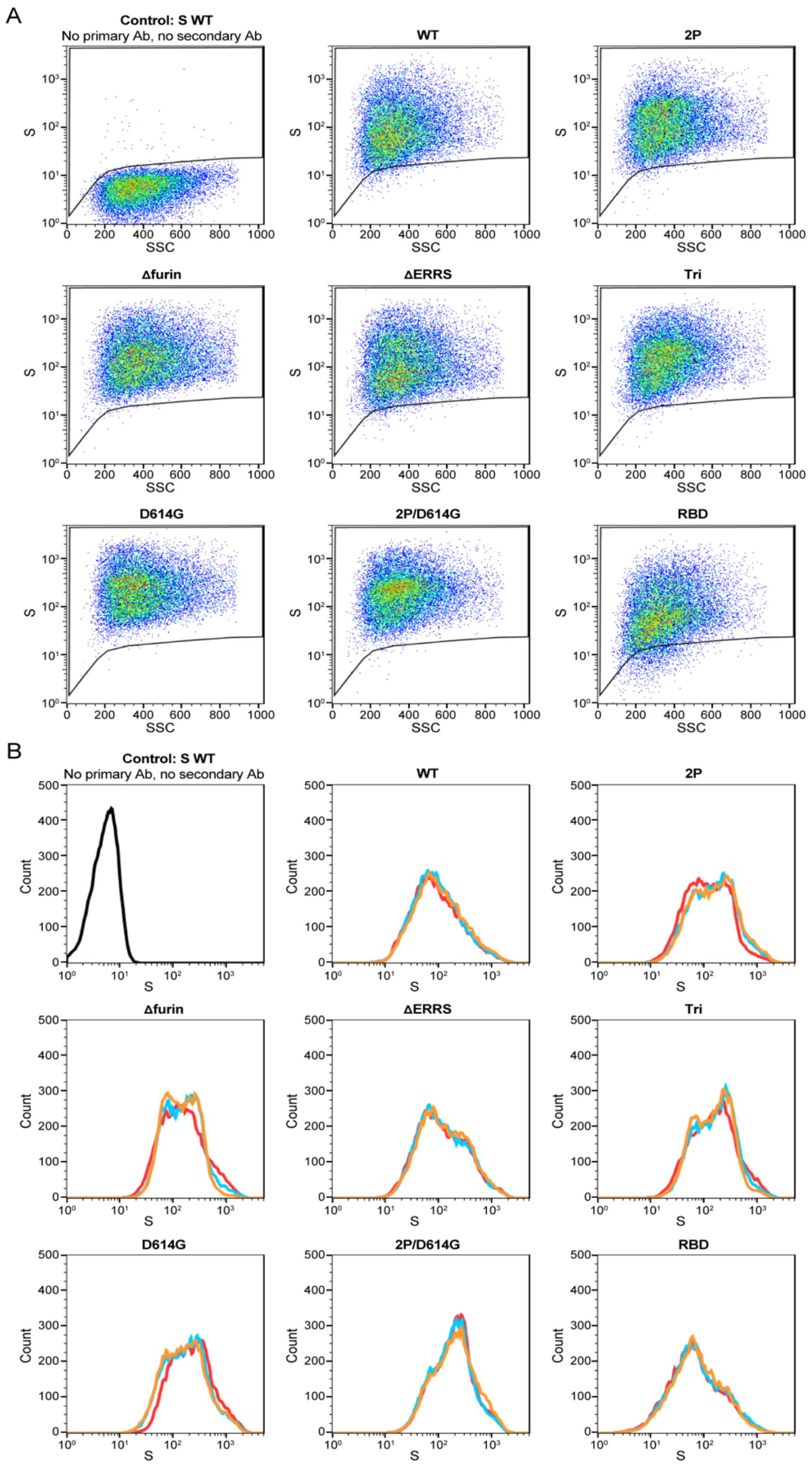

Fig. S2. Scatter plots and histograms showing anti-RBD antibody binding to rMVA-infected unpermeabilized HeLa cells related to Fig. 2D. (A) Scatter plots. (B) Histograms.

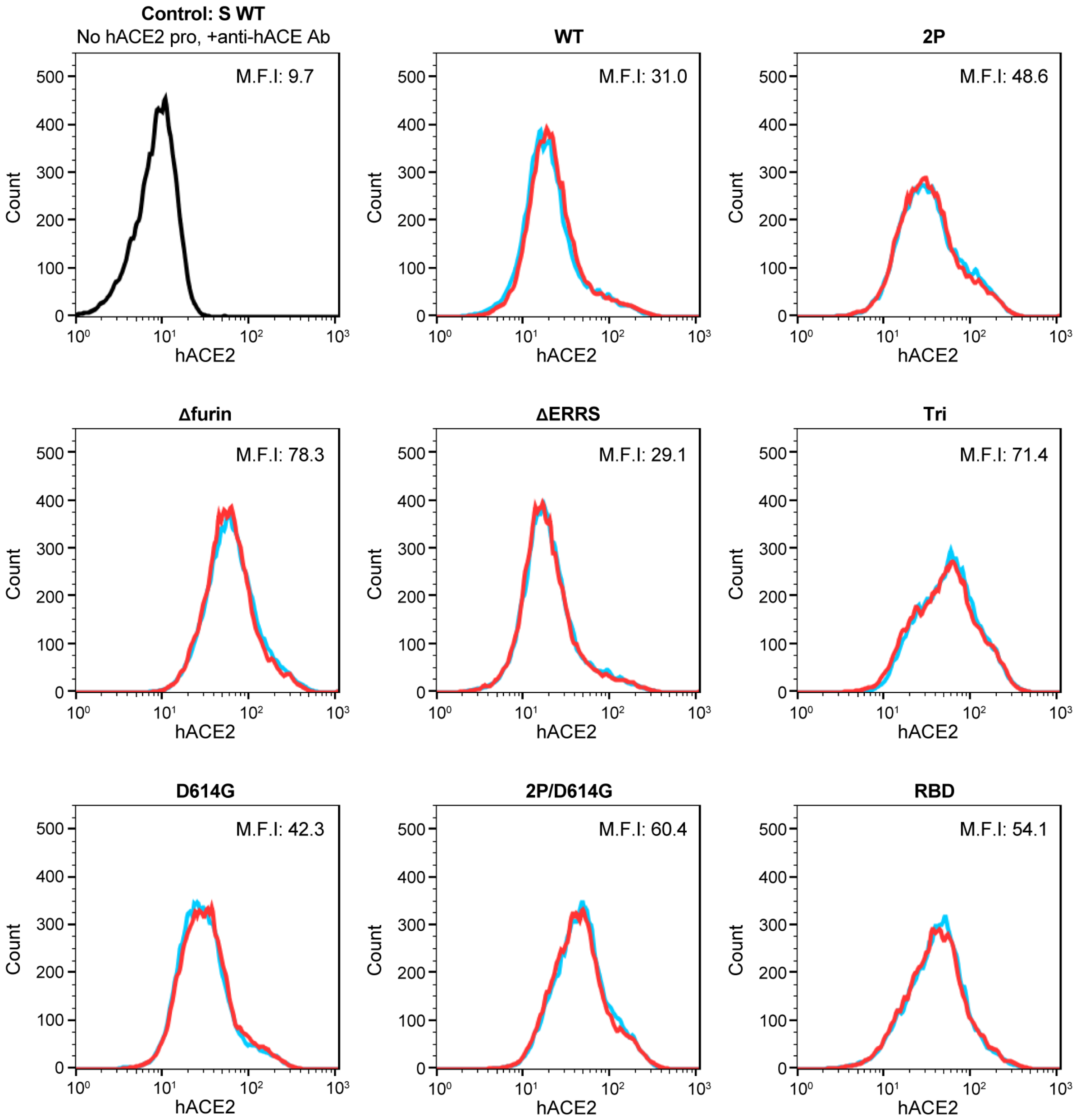

Fig. S3. Histograms showing soluble hACE2 protein binding to rMVA-infected unpermeabilized HeLa cells related to Fig. 2E.

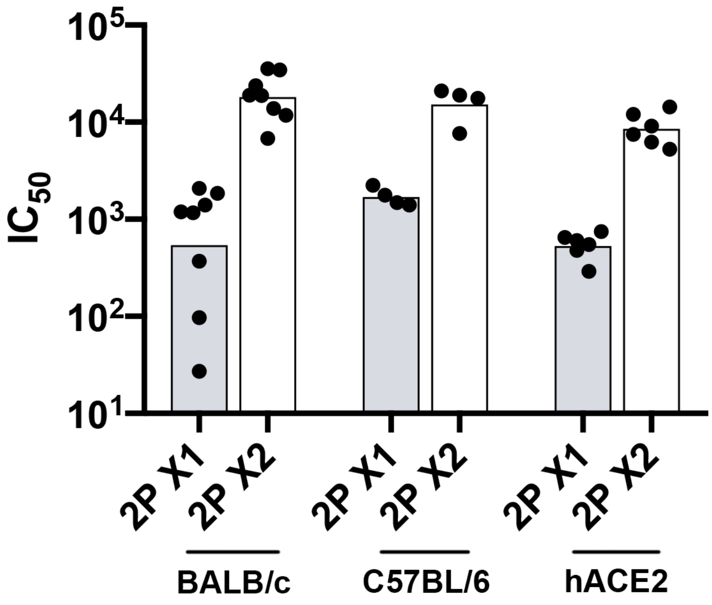

Fig. S4. Detection of MVA-neutralizing antibody after vaccination of BALB/c, C57BL/6 and K-18 hACE2 mice. Sera obtained 3 weeks after the prime (2P X1) and 2 weeks after the boost (2P X2) from the experiments described in Fig. 3 and 5 were analyzed by a semiautomated flow cytometry assay using MVA expressing green fluorescent protein. The dilutions of mouse serum that reduced the percentage of GFP-expressing cells by 50% (IC_{50}) are shown.

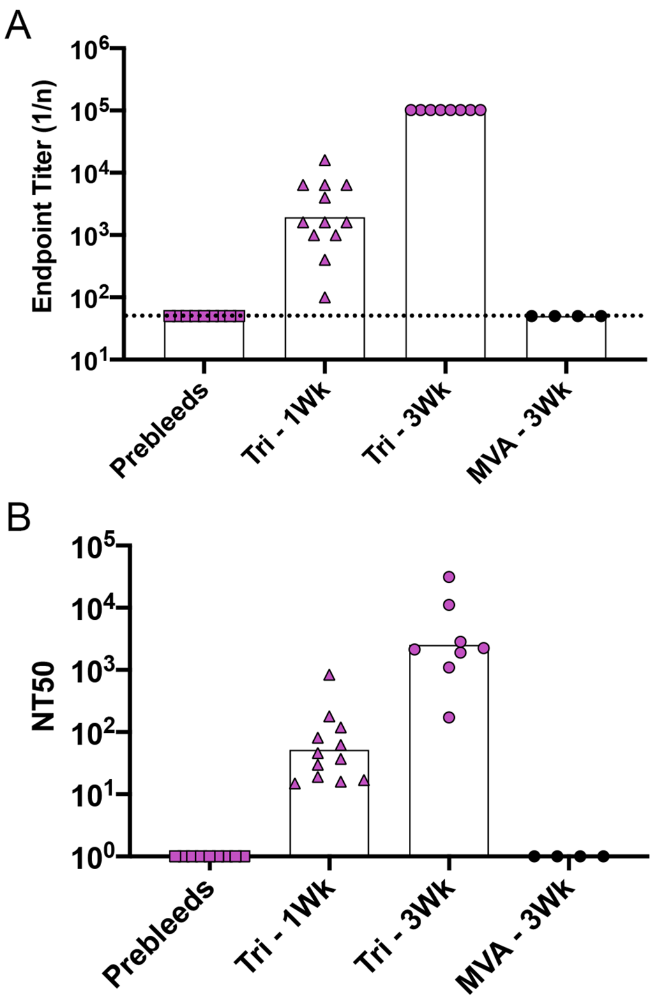

Fig. S5. Time course of antibody production. C57BL/6 mice were vaccinated with MVA or rMVA *Tri*. Serum was collected before vaccination (Prebleeds) and 1 and 3 weeks after vaccination. **(A)** S-binding antibody determined by ELISA. **(B)** Neutralizing antibody determined by pseudovirus assay.

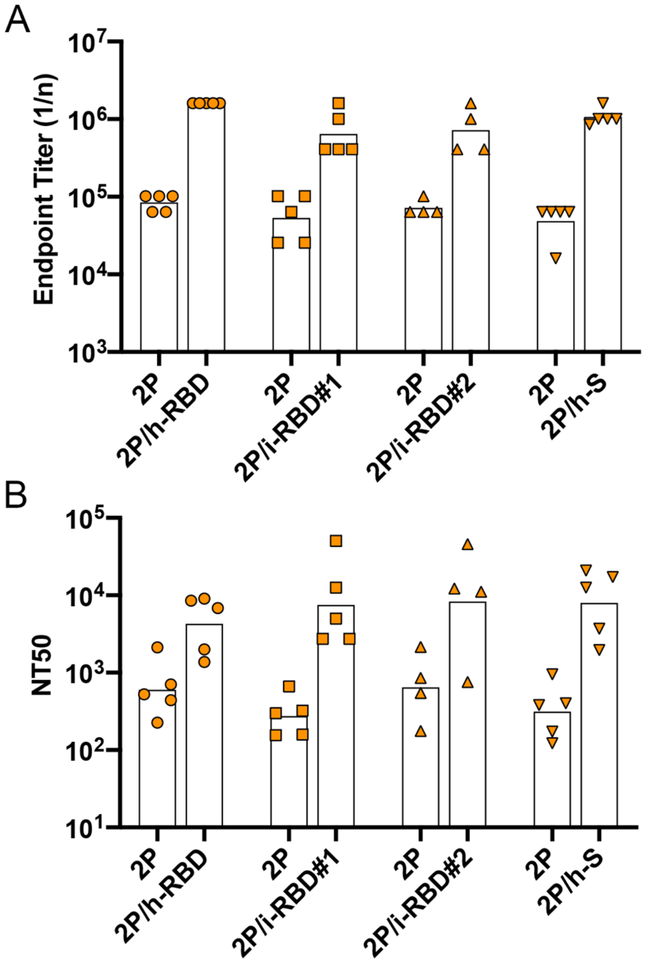

Fig. S6. Comparison of protein boosts. C57BL/6 mice were primed by IM injection with 2 X 10^7 PFU of rMVA *2P*. After 3 weeks, the mice were boosted by IM injection with 10 µg of RBD produced in human cells (h-RBD, Genscript), baculovirus produced RBD (RBD#1, Sino Biological), baculovirus produced RBD (RBD#2, provided by Eugene Valkov, NCI), or soluble S protein produced in human cells (h-S, Sino Biological). Each protein was administered with 15 µg of QS21 adjuvant. The mice were bled after 2 weeks and binding antibody and neutralization titers determined by ELISA **(A)** or pseudovirus assay **(B)**, respectively.

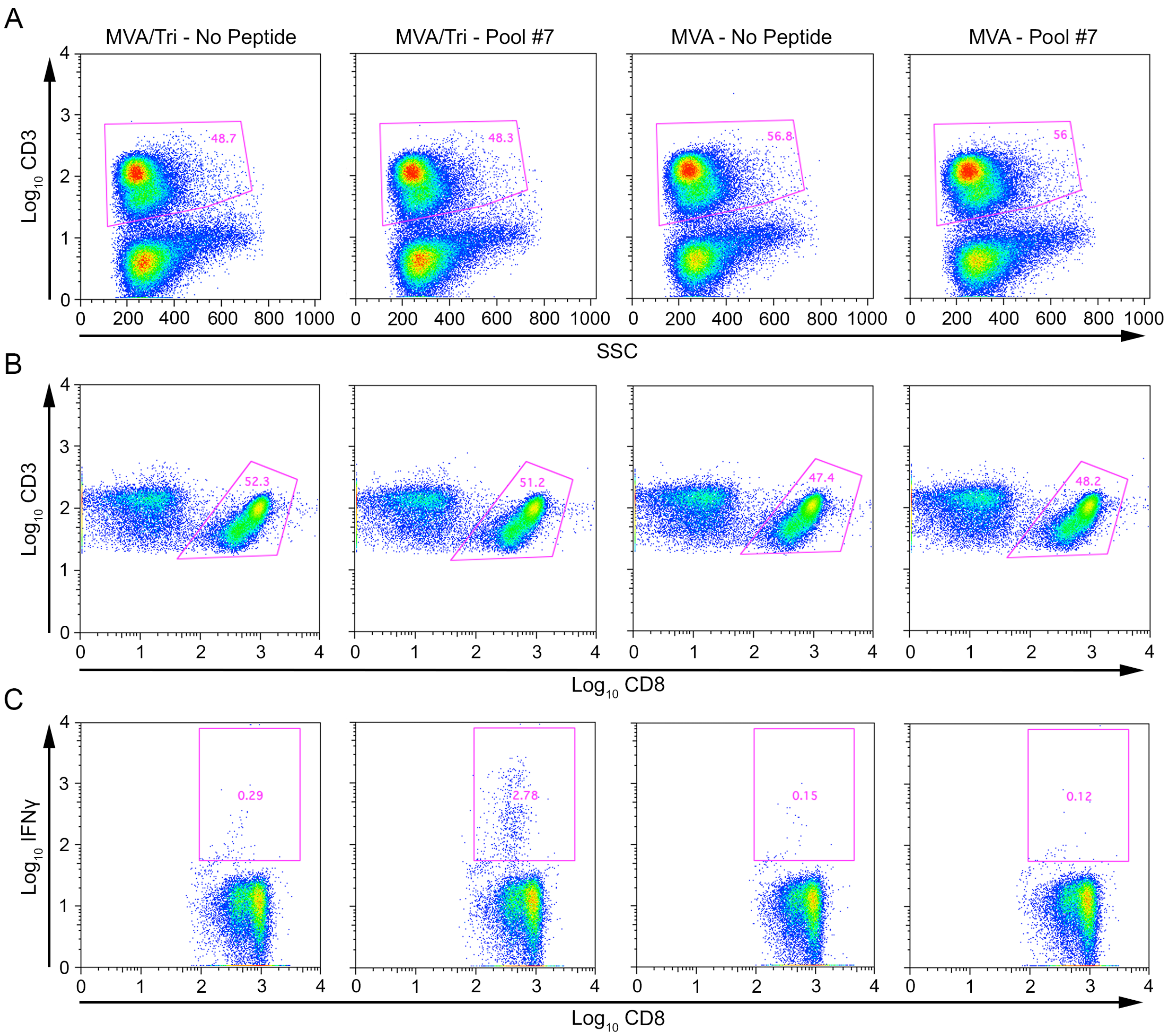

Fig. S7. Scatter plots illustrating gating strategy for detecting CD3+CD4+IFNγ+ T cells associated with Fig. 4. Spleen cells from BALB/c mice that had been primed and boosted with rMVA *Tri* or parental MVA were incubated with no peptide or peptides from pool 7. Cells were stained and analyzed as in Fig. 4.

SI References

1. L. S. Wyatt, P. L. Earl, B. Moss, Generation of Recombinant Vaccinia Viruses. *Curr. Protoc. Mol. Biol.* **117**, 16 17 11-16 17 18 (2017).
2. A. O. Hassan *et al.*, A Single-Dose Intranasal ChAd Vaccine Protects Upper and Lower Respiratory Tracts against SARS-CoV-2. *Cell* **183**, 169-184 e113 (2020).
3. P. L. Earl, J. L. Americo, B. Moss, Natural killer cells expanded in vivo or ex vivo with IL-15 overcomes the inherent susceptibility of CAST mice to lethal infection with orthopoxviruses. *PLoS Pathog* **16**, e1008505 (2020).
4. K. S. Corbett *et al.*, SARS-CoV-2 mRNA vaccine design enabled by prototype pathogen preparedness. *Nature* 10.1038/s41586-020-2622-0 (2020).
5. P. L. Earl, J. L. Americo, B. Moss, Development and use of a vaccinia virus neutralization assay based on flow cytometric detection of green fluorescent protein. *J. Virol.* **77**, 10684-10688 (2003).
